# Nuclear morphometry and chromatin texture changes in hepatocellular carcinoma samples may predict outcomes of liver transplanted patients

**DOI:** 10.1186/s12876-022-02262-5

**Published:** 2022-04-15

**Authors:** Jordan Boeira dos Santos, Rodrigo Tzovenos Starosta, Emily Ferreira Salles Pilar, Jefferson Daniel Kunz, Joelson Tomedi, Carlos Thadeu Schmidt Cerski, Rúbia Denise Ruppenthal

**Affiliations:** 1 Graduate Program in Science in Gastroenterology and Hepatology, Universidade Federal do Rio Grande do Sul, Porto Alegre, RS Brazil; 2 Department of Pediatrics, Saint Louis Children’s Hospital, Washington University, Saint Louis, MO USA; 3 Experimental Research Center, Hospital de Clínicas de Porto Alegre, Porto Alegre, RS Brazil; 4 Department of Surgical Pathology, Hospital de Clínicas de Porto Alegre, Porto Alegre, RS Brazil

**Keywords:** Hepatocellular carcinoma, Morphometry, Chromatin texture, Liver transplant

## Abstract

**Background:**

Nuclear changes are typical in the carcinogenesis of hepatocellular carcinoma (HCC). Morphometry and chromatin texture analysis are quantitative methods for their quantification. In this study, we analyzed nuclear morphometry and chromatin texture parameters in samples of hepatocellular carcinoma from liver transplant patients and their associations with clinicopathologic variables.

**Methods:**

Samples of HCC and adjacent tissue from 34 individuals were collected in tissue microarray blocks. Stained slides were microphotographed using an optical microscope and nuclear parameters analyzed in ImageJ (FracLac plug-in). ROC curve analysis was used to find accurate cut-offs for differentiation of neoplastic and non-neoplastic cells. The inter-rater agreement was also evaluated.

**Results:**

Nuclear morphometric and textural differences were observed between the samples of HCC and adjacent tissue of liver transplant patients. Lower mean gray value (*p* = 0.034) and Feret diameter (*p* = 0.024) were associated with higher Model for End-Stage Liver Disease (MELD) scores. Nuclei with larger area (*p* = 0.014) and larger Feret diameter (*p* = 0.035) were associated with lower survival. Lower aspect ratio was associated with HCC recurrence after the transplant (*p* = 0.048). The cut-off of 1.13 μm (*p* =  < 0.001) for aspect ratio and cut-off of 21.15 μm (*p* = 0.038) for perimeter were established for the differentiation of neoplastic and non-neoplastic cells. The morphometric analysis was reproducible to area, circularity, Feret diameter, mean gray value and aspect ratio between observers (*p* =  < 0.001).

**Conclusions:**

Nuclear morphometric differences between the HCC and the adjacent tissue samples were associated with prognostic variables (MELD scores, recurrence and survival) and may predict liver transplant patients’ outcomes.

## Background

Liver cancer is the third leading cause of cancer-related death worldwide, with hepatocellular carcinoma (HCC) being the most common primary histological type, corresponding to 75–85% of the cases [1]. Among the therapeutic options for HCC, liver transplantation (LT) stands out for its high capacity of inducing remission, allowing that, in a single surgical procedure, both the tumor mass and the adjacent compromised tissues are removed [2]. Regardless of the selection criteria used, approximately 15%–20% of individuals have post-LT recurrence of HCC, a factor that constitutes a significant cause of death in these patients [3].

Hepatocarcinogenesis is complex and involves genetic and epigenetic aspects that trigger malignant changes in hepatocytes [4–6]. The misstructuring of the spatial arrangement and other nuclear components are the main triggers for the modifications in the nuclear architecture of neoplastic cells that differentiate them from a healthy hepatocyte [7]. Changes in chromatin texture are frequent in tumor cells and may be associated with disease progression [8]. The investigation of these nuclear modifications has already been initiated in different types of neoplasms, including HCC [9–11], melanoma [12], lung squamous cell carcinoma [13], and basal cell carcinoma [14], all of which compare neoplastic and healthy nuclei. However, we are not aware of the use of digital analysis in the investigation of nuclear alterations in a cohort such as the one in this study, HCC samples from liver transplantation patients.

Methods for digital histological analysis have been subject to significant technological advances in the last years. A rapid evolution of computational tools can be identified in addition to the increasing complexity of algorithms [9]. Morphometry is a method capable of describing data of quantitative nature related to the area and format of a given object both at microscopic and macroscopic levels [15]. The incorporation of technological resources into nuclear morphometric analysis can assist the pathologist in discriminating and quantifying subtle characteristics that may not be noticed by a subjective analysis [16].

This study aims to identify differences in nuclear morphometry and chromatin texture in HCC samples from liver transplant patients and to assess potential associations of these differences with clinicopathologic variables of diagnostic and prognostic relevance.

## Methods

### Patients and tissues

**Fig. 1:**
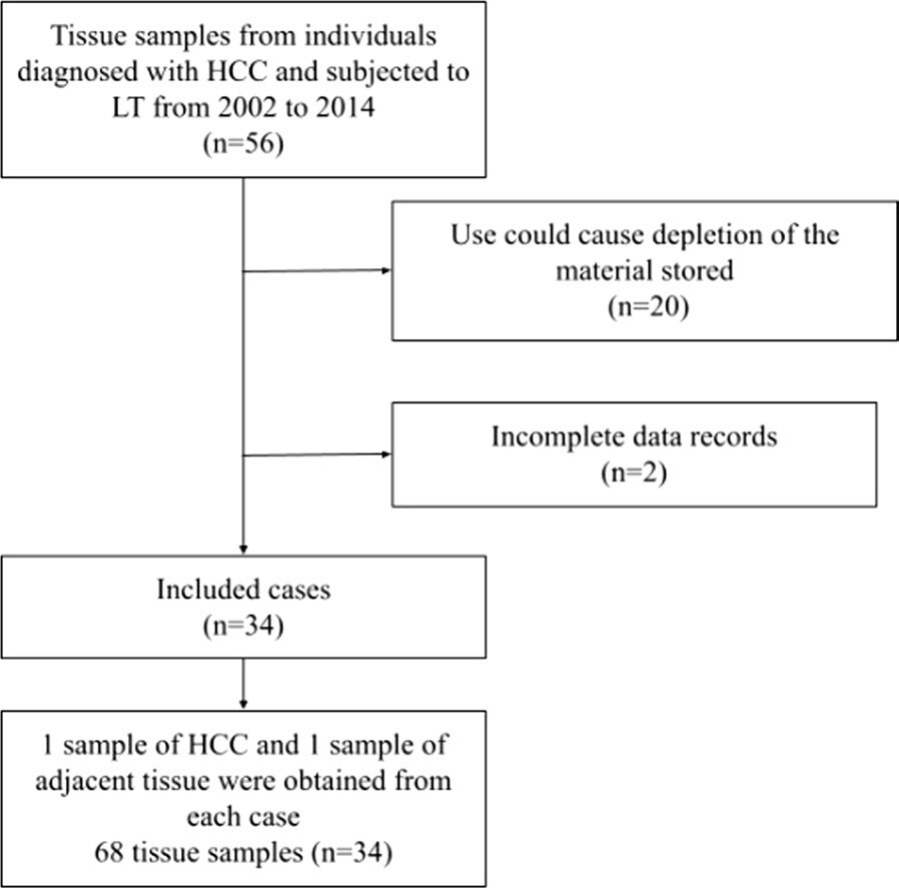
Flow chart showing the samples selection process. HCC, hepatocellular carcinoma; LT, liver transplant

The tissue samples from 34 individuals diagnosed with HCC and subjected to LT from 2002 to 2014 were included in this study. Out of these, 19 (55.9%) were male and 15 (44.1%) were female, with a mean age of 58.3 ± 8.9 years (range 17–69 years). As for the etiological factor, 23 (67.6%) individuals had a history of hepatitis C and five (14.7%) had concomitant hepatitis C and chronic alcohol abuse (Table 1). Clinicopathological data were collected from electronic medical records—gender, age, number and size of tumors, nuclear grade, Model for End-Stage Liver Disease (MELD), recurrence, vascular invasion, death, and 5-year survival—, followed by retrieval of paraffin blocks and archived slides at the Department of Surgical Pathology at the Hospital de Clínicas de Porto Alegre (HCPA). MELD score is calculated using serum bilirubin, serum creatinine, and International Normalized Ratio (INR) and is given by the formula 9.57 × loge (creatinine) + 3.78 × loge (total bilirubin) + 11.2 × loge (INR) + 6.43 [17]. In total, 20 samples were excluded from the study because their use could cause depletion of the material stored; two cases were excluded due to incomplete data records (Fig. 1). This material was analyzed by a liver pathology expert (CTSC) to confirm the diagnosis of HCC and to mark the exact location of the tumor in the investigated sample. A sample of HCC and a sample of adjacent tissue were obtained from each case, resulting in a total of 68 tissue samples included in the study. This study was approved by the HCPA Research Ethics Committee under the number #18–0551. Research consent was waived by the HCPA Research Ethics Committee due to the retrospective nature of the analyses.

**Table 1:** Clinicopathological data

Characteristic	All (n = 34)
Age < 60 years, n (%)	17 (50)
Male, n (%)	19 (55.9)
Underlying liver disease, n (%)	
Hepatitis B	2 (5.9)
Hepatitis C	23 (67.6)
Alcohol + HCV	5 (14.7)
Others	4 (11.8)
Nodule size, n (%)	
< or = 3 cm	23 (67.6)
> 3 cm	11 (32.4)
Number of tumors, n (%)	
< or = 3	32 (94.1)
> 3	2 (5.9)
Nuclear grade, n (%)	
1	2 (5.9)
2	18 (52.9)
3	9 (26.5)
Missing	5 (14.7)
MELD score, n (%)	
< 20	23 (67.6)
> 20	5 (14.7)
Missing	6 (17.6)
Recurrent, n (%)	4 (11.8)
Vascular invasion, n (%)	13 (38.2)
Death, n (%)	12 (35.3)

*n* frequency, *HCV* hepatitis C virus, *cm* centimeter, *MELD* model for end-stage liver disease

### Processing of histological material

Sample areas representing HCC or adjacent tissue were used from each individual for preparing tissue microarray (TMA) using the T-Sue system (Simport® Scientific, Beloeil, Canada). Two 2.0 mm cylinders were punctured from each original block and transferred to the receptor TMA blocks according to Kononen et al. [18]. This resulted in six TMAs, three of them containing 34 samples of tumor tissue in duplicate and three with 34 samples of adjacent tissue in duplicate. The TMAs prepared were submitted to microtomy, obtaining two sections of three μm from each block, which were arranged on histological slides and stained with hematoxylin–eosin (H&E) according to the protocols of the Department of Surgical Pathology at Hospital de Clínicas de Porto Alegre.

### Imaging

Images were captured at a resolution of 2560 × 1920 pixels using an optical microscope (OLYMPUS BX51, Ontario, Canada) with an attached camera (OLYMPUS Q-color 5 RTV, Ontario, Canada) using Q-capture Pro 7 software (https://www.photometrics.com/support/download/qcapture-pro-7) at a magnification of 1000x (oil immersion), and saved in RGB color using the .tiff file format. All images were obtained under the same light conditions and at the same microscope (Table 2). For digital analysis, one image of each case was selected, with the chosen representative region containing a minimum of 20 neoplastic (nuclei belonging to malignant cells) or hepatocellular adjacent nuclei (non-neoplastic nuclei belonging to hepatocytes) with well-demarcated sharp nuclear boundaries.

**Table 2:** Image analysis settings

Settings	Values
Dimensions	2560 × 1920 pixels
Bit intensity	24
Exposure:	100 ms
Horizontal resolution:	96dpi
Vertical resolution	96dpi
Photometric interpretation	RGB format

*ms* milliseconds, *dpi* dots per in

### Morphometry

**Fig. 2:**
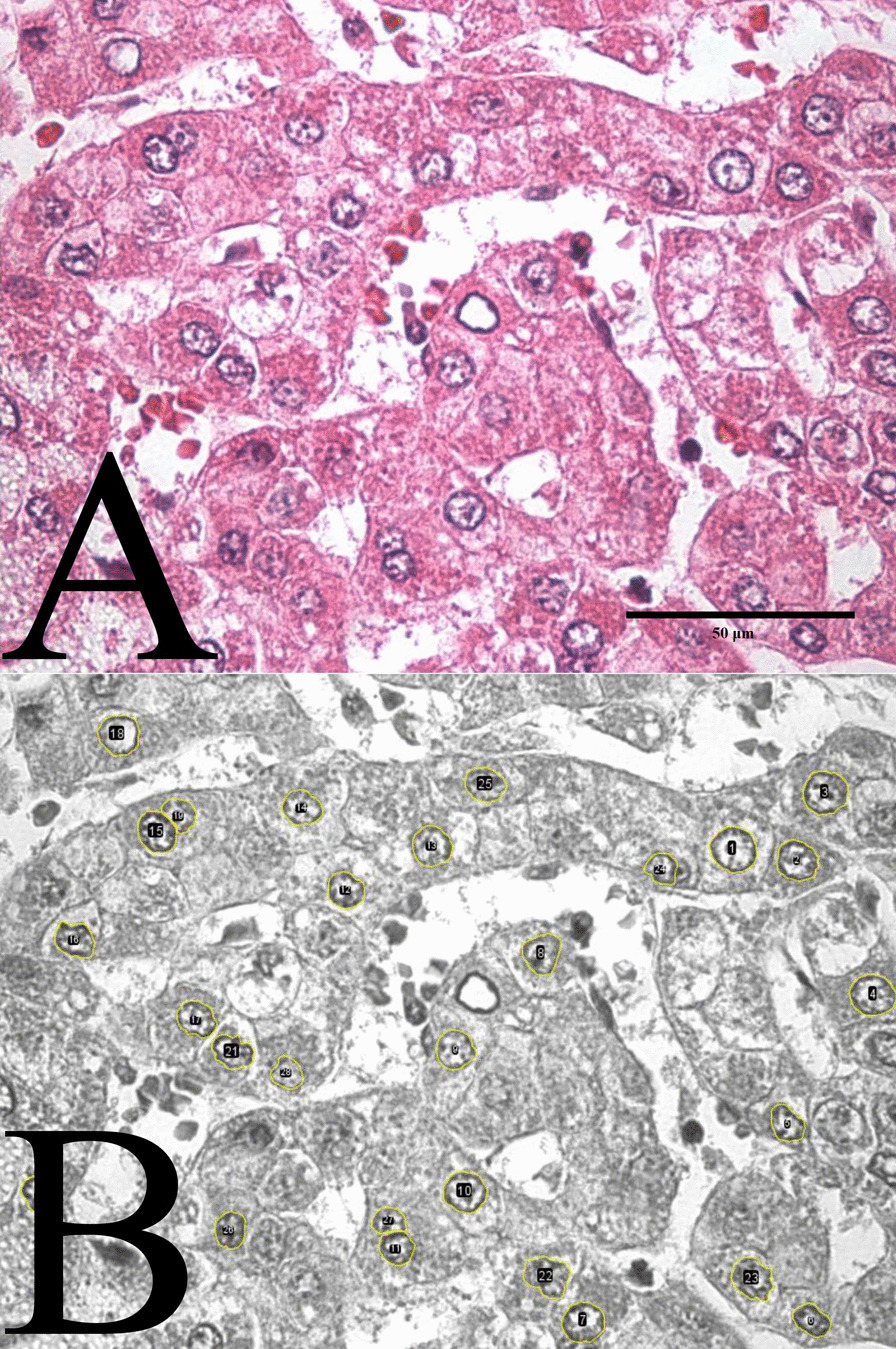
Hepatocytes of tumor tissue. **A** RGB photomicrograph (1000X). **B** Transformation image from RGB color to 8-bit and selected nucleus (1000X)

The morphometric analysis started with the manual selection of the nuclei present in the images by two researchers (JBS and RTS) who were blinded to the patient’s identity and diagnosis (HCC or adjacent tissue). Average time to manually select 20 cores was approximately 40 min per case. Each researcher selected a total of 1,548 nuclei from the tumor tissue and 988 nuclei from the adjacent tissue, corresponding to all hepatocellular or neoplastic nuclei in the images. Nuclei of overlapping cells without sharp nuclear boundaries were excluded. Afterwards, images were converted from the native RGB format to 8-bit in the ImageJ version 1.53c (https://imagej.nih.gov/ij/download.html) [19] (Fig. 2).

The following nuclear parameters were analyzed with ImageJ: area (μm^2^), perimeter (μm), circularity, Feret diameter (μm), mean gray value (MGV), solidity, aspect ratio (AR: major/minor axes) [20], and fractal dimension (FD) of the nuclear chromatin, the latter obtained with the plugin FracLac (https://imagej.nih.gov/ij/plugins/fraclac/fraclac.html). Grayscale fractal dimension was calculated by volumetric box-counting using gray value as a third dimension (pseudo-axis). Feret diameter is defined as the mean measure of the projection of an object to orthogonal tangential axes [21].

MGV was corrected (corrected MGV, cMGV) with the formula cMGV = 255-MGV to eliminate possible artificial differences caused by staining irregularities. To normalize the nuclear cMGV, six areas of hepatocellular cytoplasm were selected in each image and the mean cytoplasmic MGV was considered as representative of the background value. This was used to calculate the ncMGV (normalized corrected MGV) by subtracting the gray value measured in the background regions (ncMGV = cMGV − background cMGV).

### Statistical analysis

Statistical analysis was performed with the Statistical Package for Social Sciences (SPSS) version 18.0 (SPSS Inc., Chicago, USA). The Shapiro–Wilk test was used to assess normality. All results were expressed as mean ± standard deviation for continuous variables and frequencies for categorical variables. The parametric paired Student’s t-test was used to compare morphometric values between tumor tissue and adjacent tissue. For comparing values between the different clinical and pathological variables of the tumor, the independent-samples Student’s t-test or one-way analysis of variance (ANOVA) were used. A post-hoc test (e.g.Bonferroni test) was not performed due to the small sample size. The sensitivity, specificity, and area under the receiver operator characteristic (ROC) curve (AUC) were calculated for each parameter to determine the validity of the morphometric method. Values of *p* < 0.05 were considered statistically significant. Furthermore, regression analysis was performed with Pearson correlation in order to test the reproducibility of the morphometric analysis, according to the results obtained by manual selection of nuclei by the two blinded researchers, as described above.

## Results

### Nuclear morphometry and chromatin texture differences between HCC and adjacent tissue

A difference between the HCC and the adjacent tissue samples was found in perimeter (*p* = 0.025), circularity (*p* =  < 0.001), solidity (*p* =  < 0.001), AR (*p* =  < 0.001), and FD (*p* = 0.001), and the difference in relation to the texture of nuclear chromatin, differences were also found in the FD between the samples of HCC and adjacent tissue (*p* = 0.001) as shown in Table 3.

**Table 3:** Morphometric and chromatin texture characteristics

Characteristic	Tumor—mean (Standard deviation)	Adjacent—mean (Standard deviation)	p*
*Nuclear shape descriptors*			
Area (µm^2^)	48.65 ± 14.90	53.51 ± 7.13	0.066
Perimeter (µm)	26.08 ± 4.06	24.07 ± 2.22	0.025
Circularity	0.833 ± 0.04	0.955 ± 0.01	< 0.001
Feret (µm)	85.33 ± 14.78	89.80 ± 5.69	0.094
ncMGV (µm)	22.64 ± 9.83	25.18 ± 6.33	0.206
Solidity	0.982 ± 0.009	0.995 ± 0.002	< 0.001
AR	1.28 ± 0.08	1.15 ± 0.06	< 0.001
*Chromatin texture descriptor*			
Fractal dimension	1.16 ± 0.03	1.19 ± 0.02	0.001

*p, statistical significance; *µm* micrometer, *ncMGV* normalized corrected mean grey value, *AR* aspect ratio

### Nuclear morphometry association with clinicopathological and prognostic variables

Feret diameters in the HCC samples varied according to age groups (*p* = 0.034), being higher in individuals aged 60 years or over. Significant differences were found in MELD scores in relation to ncMGV (*p* = 0.034) and Feret diameter (*p* = 0.024), both parameters being lower in individuals with MELD scores above 20 points. Regarding survival, nuclei with higher measurements of area (*p* = 0.014) and Feret diameter (*p* = 0.035) were found in individuals who had a post-transplant survival time shorter than 5 years. The AR measurement differed between the groups in relation to the recurrence of the HCC after the transplant (*p* = 0.048) with lower values among the individuals who relapsed (Table 4).

**Table 4:** Comparison between nuclear parameters and clinical-pathological characteristics

Characteristic	Area(µm^2^) mean ± SD	p*	Perimeter(µm) mean ± SD	p*	Circularity mean ± SD	p*	Feret(µm) mean ± SD	p*	ncMGV(µm) mean ± SD	p*	Solidity mean ± SD	p*	AR mean ± SD	p*	FD mean ± SD	p*
Sex																
Male	49.84 ± 15.30	0.606	26.92 ± 4.02	0.175	0.834 ± 0.04	0.868	87.36 ± 13.70	0.375	22.27 ± 9.97	0.812	0.981 ± 0.009	0.796	1.26 ± 0.09	0.212	1.17 ± 0.03	0.251
Female	47.13 ± 1477		25.00 ± 3.98		0.823 ± 0.04		82.76 ± 16.16		23.10 ± 9.97		0.182 ± 0.009		1.31 ± 0,07		1.15 ± 0.03	
Age (years)																
< 60	44.68 ± 15.63	0.123	25.68 ± 4.62	0.580	0.837 ± 0.04	0.280	80.01 ± 14.46	0.034	20.11 ± 10.4	0.136	0.980 ± 0.007	0.326	1.27 ± 0.09	0.879	1.17 ± 0.03	0.379
= or > 60	52.61 ± 13.44		26.47 ± 3.50		0.821 ± 0.04		90.65 ± 13.47		25.16 ± 8,7		0.983 ± 0.010		1.28 ± 0.08		1.16 ± 0.03	
Number tumor																
< or = 3	48.19 ± 14.94	0.486	26.01 ± 4.09	0.723	0.837 ± 0.04	0.059	85.12 ± 15.04	0.750	23.01 ± 9.85	0.390	0.982 ± 0.009	0.694	1.28 ± 0.08	0.614	1.16 ± 0.03	0.302
> 3	55.91 ± 17.21		27.09 ± 4.58		0.769 ± 0.06		88.64 ± 13.13		16.74 ± 10.34		0.979 ± 0.007		1.25 ± 0.12		1.14 ± 0.05	
Nodule size (cm)																
< or = 3	48.97 ± 11.68	0.884	25.55 ± 3.02	0.385	0.828 ± 0.04	0.859	84.46 ± 14.35	0.628	26.70 ± 1.13	0.784	0.983 ± 0.009	0.379	1.27 ± 0.08	0.507	1.15 ± 0.03	0.128
> 3	47.97 ± 20.79		27.19 ± 5.68		0.834 ± 0.02		87.14 ± 16.21		23.32 ± 9.59		0.980 ± 0.009		1.34 ± 0.10		1.18 ± 0.03	
Nuclear grade																
1	58.36 ± 14.18	0.757	27.52 ± 3.45	0.842	0.848 ± 0,02	0.796	94.57 ± 11.27	0.733	30.50 ± 18.15	0.064	0.996 ± 0.001	0.088	1.17 ± 0.01	0.145	1.13 ± 0.03	0.435
2	51.33 ± 13.32		26.25 ± 3.32		0.821 ± 0.04		87.25 ± 14.48		23.89 ± 10.48		0.980 ± 0.008		1.29 ± 0.08		1.16 ± 0.06	
3	50.38 ± 14.44		25.90 ± 3.96		0.843 ± 0.05		85.07 ± 17.57		19.33 ± 6.30		0.981 ± 0.011		1.30 ± 0.09		1.15 ± 0.03	
MELD																
< 20	50.99 ± 15.92	0.200	27.02 ± 4.06	0.066	0.828 ± 0.04	0.636	89.50 ± 14.89	0.024	23.84 ± 9.61	0.034	0.982 ± 0.009	0.975	1.28 ± 0.09	0.499	1.16 ± 0.03	0.279
> 20	41.42 ± 4.44		23.44 ± 1.21		0.834 ± 0.04		73.17 ± 4.69		13.58 ± 7.13		0.982 ± 0.002		1.31 ± 0.08		1.14 ± 0.04	
Recurrent																
Absent	48.53 ± 15.29	0.908	26.14 ± 4.19	0.802	0.823 ± 0.04	0.957	85.39 ± 15.40	0.951	21.92 ± 9.68	0.248	0.982 ± 0.009	0.921	1.29 ± 0.08	0.048	1.16 ± 0.03	0.946
Present	49.48 ± 13.50		25.59 ± 3.36		0.971 ± 0.02		84.89 ± 10.48		28.04 ± 10.59		0.982 ± 0.009		1.21 ± 0.08		1.16 ± 0.03	
Vascular inv																
Absent	48.23 ± 11.07	0.862	25.34 ± 2.81	0.256	0.827 ± 0.04	0.469	84.18 ± 13.80	0.574	23.10 ± 9.74	0.736	0.984 ± 0.009	0.123	1.29 ± 0.09	0.334	1.16 ± 0.03	0.504
Present	49.31 ± 20.14		27.26 ± 5.44		0.834 ± 0.03		87.18 ± 16.66		21.90 ± 10.32		0.978 ± 0.009		1.26 ± 0.07		1.16 ± 0.03	
Death																
No	46.91 ± 16.23	0.657	26.10 ± 4,48	0.829	0.830 ± 0.03	0.339	86.02 ± 16.28	0.836	21.62 ± 9.52	0.475	0.983 ± 0.008	0.658	1.30 ± 0.08	0.290	1.16 ± 0.03	0.892
Yes	50.21 ± 12.48		25.87 ± 3.39		0.828 ± 0.05		84.60 ± 11.43		24.30 ± 11.67		0.981 ± 0.011		1.25 ± 0.09		1.16 ± 0.03	
Survival (years)																
< 5	55.32 ± 14.10	0.014	27.08 ± 3.61	0.273	0.821 ± 0.04	0.132	92.84 ± 14.40	0.035	22.09 ± 9.02	0.076	0.984 ± 0.008	0.412	1.29 ± 0.09	0.440	1.16 ± 0.04	0.923
> 5	39.52 ± 10.50		24.71 ± 4.26		0.843 ± 0.03		76.52 ± 8.12		23.35 ± 11.99		0.980 ± 0.009		1.27 ± 0.07		1.16 ± 0.03	

*p, statistical significance; *SD* standard deviation, *µm* micrometer, *cm* centimeter, *ncMGV* normalized corrected mean grey value, *AR*, aspect ratio, *FD* fractal dimension, *MELD* Model End-Stage Liver Disease, *Vascular inv*. Vascular invasion

### Diagnostic validation of the nuclear morphometry analysis

**Fig. 3:**
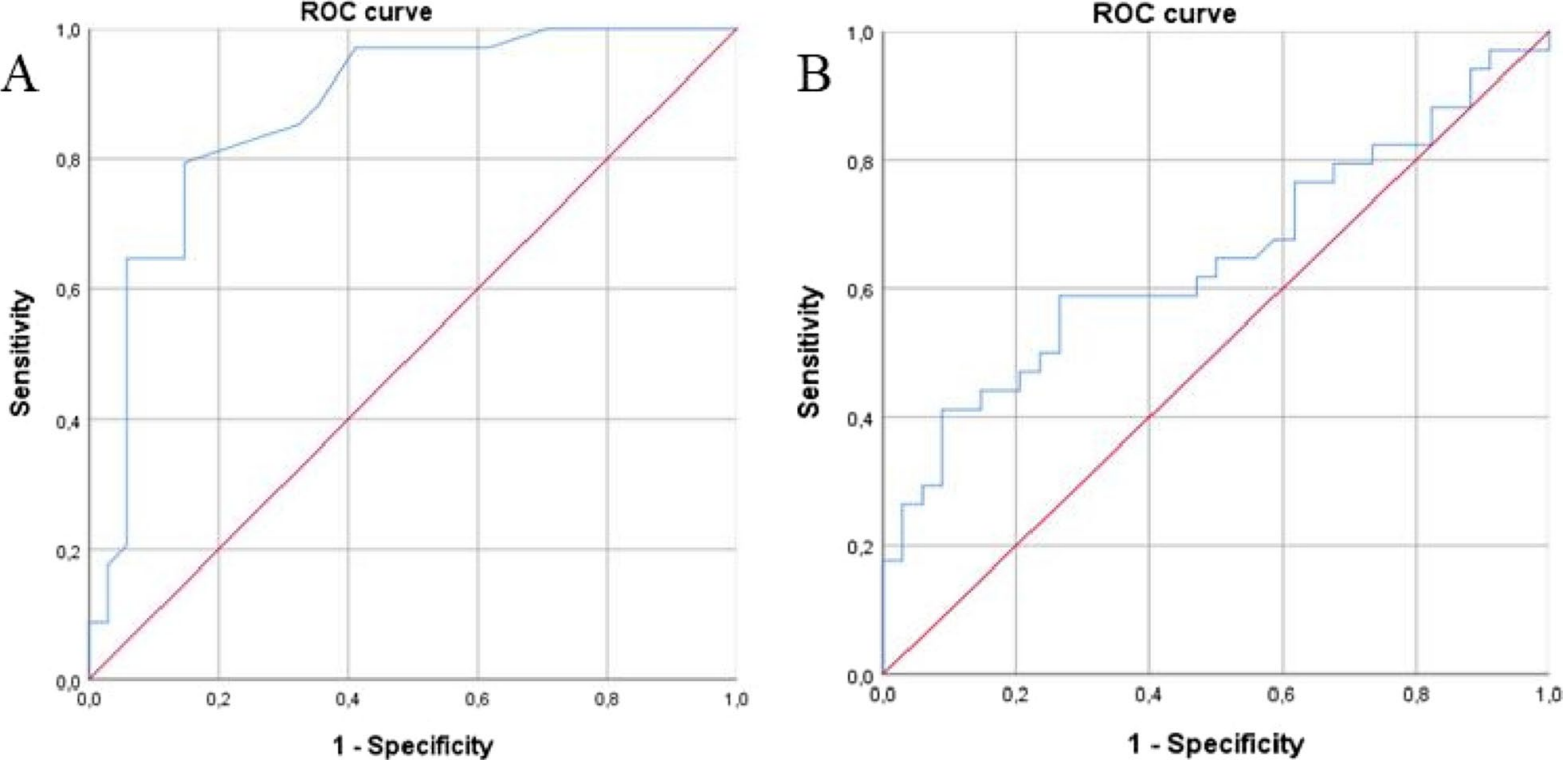
ROC curve of **A** aspect ratio and **B** nuclear perimeter, which represents the overall diagnostic value of the model in predicting the presence of cell malignancy

A  ROC curve analysis of the multiple parameters evaluated was carried out in order to validate the use of morphometry in the diagnosis of HCC. The AR cut-off point of 1.13 μm has a sensitivity of 97% and specificity of 70%, and AUC of 0.87 (*p* =  < 0.001, 95% confidence interval = 0.78–0.95) for discrimination of neoplastic cells (Fig. 3A). The nuclear perimeter cut-off point of 21.15 μm has a sensitivity of 94%, specificity of 82%, and AUC of 0.64 (*p* = 0.038, 95% confidence interval 0.51–0.78) for discrimination of neoplastic cells (Fig. 3B). Other parameters were not significant in the ROC curve analysis.

### Inter-observer concordance

Some parameters were shown to be influenced by subjectivity in nuclear segmentation (marking of nuclei), performed independently by two blinded researchers. The results obtained by two blinded researchers were statistically significant for area, circularity, Feret diameter, ncMGV, and AR (Pearson correlation, *p* =  < 0.001). No correlation was found for perimeter (*p* = 0.114), solidity (*p* = 0.337) and FD (*p* = 0.823).

## Discussion

In this study, the differences found in the nuclear measurements of the perimeter, circularity, solidity, and AR corroborate the occurrence of irregularities in the normal morphology of hepatocytes as a result of the malignant transformation process and demonstrate the excellent potential in combining this tool with the traditional histopathological analytic method.

We showed that morphometry can be used as a tool to discriminate tumoral and adjacent normal tissues. Our data complements the results of Hassan et al. [22] who performed imaging analysis of tumoral HCC nuclei and hepatocellular nuclei from surgical tumor-free safe margins in a cohort of patients with chronic hepatitis C; in that study, a significantly lower nuclear area was observed in tumor cells and in the surgical tumor-free margin hepatocytes than in patients without HCC. These data are indeed more significant when we consider that most patients included in our study also have a pre-transplantation history of hepatitis C.

The existence of nuclear morphometric changes has already been verified in studies with different types of tumors [10, 12, 23] including studies such as the one by Mendaçolli et al. [14] in which significant changes in morphometric and chromatin texture patterns were observed between basal cell carcinoma samples and the unaffected basal epithelium. Additionally in that study, the sclerodermiform type neoplasms presented larger nuclear area and diameter in relation to nodular and superficial types, suggesting that genomic or metabolic differences would also be determinant for independent biological behavior among basal cell carcinoma subtypes.

Regarding the chromatin texture, the FD in this study was lower in the HCC samples compared to the adjacent tissues. These findings differ from those observed in the study by Gheonea et al. [9] which obtained an increased FD value in HCC when compared to that observed in adjacent hepatic tissue. A possible explanation for the disagreement of FD values in tumor tissue between the studies may be related to inter-rater agreement in the measurement of this parameter: the non-significance of inter-rater agreement demonstrated in our study may have influenced the outcome, both in our study and in the study by Gheonea et al. [9]. In order to make stronger conclusions from the analysis of FD, it is necessary to improve the method for its measurement, increasing its reproducibility.

Quantitative analysis is a useful tool for developing new diagnostic methods [7]. Image softwares can make possible data checking between different researchers in all the samples measured, which allows for the exclusion of manual selections [24]. The correlation of inter-rater metrics was statistically significant in most parameters assessed, indicating that these findings might be reproducible in future studies and increasing the utility of our morphometric method for clinical practice.

Our study is the first to observe the association between changes in nuclear morphology and clinically relevant variables related to determination of the prognostic of post-LT patients. Our data showed an association of survival in a time period of less than 5 years with larger area and Feret diameter nuclei, and lower nuclear measurements of some parameters (Feret diameter and ncMGV) associated with higher MELD scores. This means that these parameters may be used to help predict outcomes of liver transplantation, providing a greater scientific basis for medical decisions making that directly affect medical practice and that broaden the scope of personalized medicine in HCC.

Although Feret diameter and AR or nuclear perimeter are close indicators of nuclear irregularity. However, either the perimeter or AR did not correlate with patients’ survival after liver implantation. This apparent discrepancy is due to the fact that AR and Feret diameter, despite being both indices of nuclear irregularity, are only semi-dependent parameters that conserve independent degrees of freedom.

Lower AR values were found in individuals who had post-LT HCC recurrence. The risk assessment of post-LT HCC recurrence using pathological characteristics of the explant is an important finding as it can lead to refining of the prognostic assessment and in the future may help to delineate therapy and screening protocols [25].

A unique result of this study is the definition of cut-off values to differentiate malignant and healthy hepatocytes using the AR, which helps to establish more objective diagnostic criteria for cell differentiation. Values defined by the ROC curve related to AR are results not used in other studies. Sensitivity and specificity values for nuclear perimeter in our study are similar to the values found by Ambroise et al. [26] who showed that a cut-off level of 33.2 µm for nuclear perimeter could differentiate malignant and benign pleural effusions. However, despite the computer analysis by ImageJ following a similar methodology, they used analyses applied to effusion cytology, in addition to evaluating for each case only ten representative nuclei from ten different fields.

Our study does have some limitations. The first is the reduced number of samples, since many samples had to be excluded from the analysis due to the loss of tissue integrity caused by the prolonged storage time of the paraffin blocks, possibly hampering the power of this study. Secondly, it is known that cirrhosis, a subjacent abnormality in all cases, can affect the measurements obtained in the tissue adjacent to the tumor used in comparison with the HCC—although this does not limit the differentiation of cells from the same sample, it limits the applicability of the exact values to healthy liver parenchyma, and so further studies evaluating non-cirrhotic patients are necessary. Finally, we did not investigate the molecular events causing the observed morphometric differences in this study. Therefore, we propose that future studies incorporate the use of methods to evaluate these events such as chromatin immunoprecipitation associated with DNA sequencing for an in-depth elucidation of the mechanisms that trigger the morphometric changes observed here.

## Conclusions

In conclusion, a significant difference was found in nuclear morphometry (perimeter, circularity, solidity, and AR) and in chromatin texture (FD) between HCC and adjacent tissue hepatocytes from liver transplanted patients, as well as an association of these alterations with clinically relevant variables (age, MELD score, post-LT HCC recurrence and survival), directly involved with the definition of the patient’s prognosis post-LT. We found our morphometric analysis to be replicable between raters. We also encountered a high sensitivity and specificity in AR and nuclear perimeter for discriminating between neoplastic and non-neoplastic hepatocytes. Further studies are necessary to investigate the applicability of the quantitative analysis to elucidate mechanisms associated with the development of HCC in order to validate the diagnosis and prognosis of this tool and its future use in clinical practice.

## Data Availability

The datasets and materials generated during and/or analyzed during the current study are available from the corresponding author upon reasonable request.

## Abbreviations

AR: Aspect ratio
dpi: Dots per in
FD: Fractal dimension
HCC: Hepatocellular carcinoma
HCPA: Hospital de Clínicas de Porto Alegre
H&E: Hematoxylin & eosin
INR: International Normalized Ratio
LT: Liver transplantation
MELD: Model for End-Stage Liver Disease
MGV: Mean gray value
ms: Milliseconds
TMA: Tissue microarray
